# Does the use of health technology assessment have an impact on the utilisation of health care resources? Evidence from two European countries

**DOI:** 10.1007/s10198-020-01160-5

**Published:** 2020-02-05

**Authors:** B. Corbacho, M. Drummond, R. Santos, E. Jones, J. M. Borràs, J. Mestre-Ferrandiz, J. Espín, N. Henry, A. Prat

**Affiliations:** 1 York Trials Unit, ARRC Building, Department of Health Sciences, University of York, York, UK; 2 Centre for Health Economics, University of York, York, UK; 3 Pope Woodhead, London, UK; 4 Department Clinical Sciences, Universidad de Barcelona, Barcelona, Spain; 5 Independent Economics Consultant, Madrid, Spain; 6 Escuela Andaluza de Salud Publica, Investigación Biosanitaria (ibs.GRANADA), Granada, Spain; 7 CIBER de Epidemiología y Salud Pública (CIBERESP), Madrid, Spain; 8 IQVIA, London, UK; 9 Pharmacy and Medicines Department, Servei Catala` de la Salut (CatSalut), Barcelona, Spain

**Keywords:** HTA methods, Decision making, Cost-effectiveness, Cancer, I15, I18

## Abstract

**Objectives:**

A centralised approach to health technology assessment (HTA) may facilitate optimal use of HTA resources. A regional approach may increase the chances of local implementation of recommendations. This study aimed to compare assessment procedures in England (centralised HTA approach) with Spain (regional HTA approach) discussing key challenges and opportunities from both approaches.

**Methods:**

We compared technology assessments of anticancer medicines in the two jurisdictions from 2008 to 2015. To assess the implementation of HTA recommendations, we assessed trends in medicine usage using regression methods. We used IQVIA data, from 2011 to 2016, for a sample of 11 medicines. We used CatSalut data from Catalonia to assess the implementation of local recommendations.

**Results:**

In England, 66 assessments were undertaken by the National Institute for Health and Care Excellence (NICE), using a standardised methodology. In Spain, there were 79 reports undertaken by a range of bodies using a shared process and coordinated through the GENESIS collaboration; the assessment methods used varied substantially. Overall, the recommendations in the two jurisdictions were similar. Regression analyses indicate that where there is a positive recommendation by HTA bodies, the usage of the medicine responds most strongly (p < 0.001) in Catalonia (4.892), followed by England (3.120) and Spain (1.693).

**Conclusions:**

This study suggests that medicine utilisation does respond to the positive recommendations of HTA bodies. However, if HTA capacity is organised primarily regionally, considerable effort may be required in coordination, to ensure consistent and rigorous assessments and adequate implementation of HTA findings.

**Electronic supplementary material:**

The online version of this article (10.1007/s10198-020-01160-5) contains supplementary material, which is available to authorized users.

## Introduction

Different jurisdictions organise their health technology assessment (HTA) capacity in different ways. Some establish capacity at the central level and undertake assessments that result in recommendations for the whole country; others establish capacity at the regional level resulting in several HTA bodies within the same country and the possibility of several, potentially different sets of recommendations. In principle, a centralised approach offers the potential to pool the scarce resources devoted to HTA, to conduct consistent assessments and issue national recommendations that may encourage a uniform development of services across the whole country.

At the European level, this rationale of “economies of scale” is also being supported by the recent European Commission proposal for Regulation on HTA (European Commission. Regulation of the European Parliament and of the Council on health technology assessment and amending Directive 2011/24/EU. https://ec.europa.eu/health/sites/health/files/technology_assessment/docs/com2018_51final_en.pdf (2018). (Accessed 29 Mar 2019), although strictly speaking, the proposal is only about joint clinical assessments, leaving economic evaluation as a member state competency. On the other hand, a regional approach may be more fragmented, but may help in tailoring recommendations to local needs, perhaps encouraging a greater adoption of the recommendations made.

England is an example of a country following primarily a centralised approach. Assessments of medicines and other health technologies are conducted by the National Institute for Health and Care Excellence (NICE), which follows a consistent methodology [1, 2]. Based on a review of the clinical and economic evidence, the incremental cost per quality-adjusted life-year (QALY) of the new technology is estimated and compared with NICE’s ‘threshold’ of acceptable incremental cost-effectiveness [3]. This threshold can be increased for end-of-life treatments if the criteria in the end-of life protocol are demonstrated [4], or for highly specialised technologies including medicines for ultra-rare diseases [5]. Recommendations are then issued to the National Health Service (NHS) in England, which is obliged to fund those technologies that receive positive recommendations within 3 months.

An important feature of NICE recommendations in recent years is the application of Patient Access Schemes (PAS). Under this approach, the NICE recommendation (whether ‘recommended ‘or ‘restricted’) is conditional on the manufacturer offering a PAS, which usually involves a confidential price discount.

Additionally, the Cancer Drugs Fund (CDF) has provided a complementary source of funding for those cancer medicines not having positive recommendations from NICE. It was established (as a temporary measure) by the Government in 2011 to provide expanded patient access for cancer medications. However, after exceeding its allocated budget each year since 2014, a new framework for the CDF was established in 2016 [6], to become a “managed access fund”. The new approach allows NICE to recommend medicines for use within the CDF in situations where it considers that a highly promising medicine in an area of high unmet need area satisfies the criteria for routine use in the NHS, but still has remaining uncertainty about clinical and cost-effectiveness. The final recommendation for the medicine is then issued after further evidence has been generated, generally after 2 years. The recent changes to the CDF have had a marked effect, increasing the overall approval rate of decisions recommending routine use and managed access via the CDF [7].

By contrast, Spain is an example of a country following primarily a regional HTA approach, with 17 regions having complete control of their own health budget. Nevertheless, at the central level, once a medicine is licensed (by the Agencia Espa Española de Medicamentos y Productos Sanitatios), the Ministerio de Sanidad, Consumo y Bienestar Social (MSC) decides on its reimbursement and pricing based, among other things, on an assessment of therapeutic value, although there is lack of clarity in how these assessments are done, and their impact on the decision. Importantly, there is no HTA assessment similar to NICE’s. A positive recommendation by the MSC implies that the medicine is approved for regional use and, although regions could reverse the central decision, this rarely happens.

However, hospital budgets are limited, so their pharmaco-therapeutic commissions, usually led by hospital pharmacists, often fund the medicine conditional on very specific clinical and cost criteria. The impact that economic evaluation has had in informing national and local policies within the health care systems of England and Spain differs substantially [8, 9]. In Spain, although the MSC has commissioned two proposals for methodological standardisation of economic analysis [10], there are currently no formal requirements for cost-effectiveness, although it is a criterion included in the legislation. Regardless of the methods that have been proposed by academic groups [11, 12], economic evaluation is not systematically used in health policy decision making in Spain.

This hierarchical approach in Spain has led to a complex HTA system for pharmaceuticals that involves multiple organisations and potentially multiple assessments for the same medicine at three different levels [13]. At the national level, the MSC issues Informes de Posicionamiento (IPT) reports, but these concentrate mainly on effectiveness (it can be considered as a “clinical HTA”, and similar to the proposed EU joint clinical assessment mentioned above). At the regional level, there are a number of bodies assessing pharmaceuticals both in primary and secondary care. Since 2004, hospital pharmacy units within the country have been coordinated through the Grupo de Evaluación de Novedades, EStandarización e Investigación en Selección de Medicamentos (GENESIS) collaboration, led by the Spanish Society for Hospital Pharmacy (SEHF). GENESIS shared assessments are made according to a protocol and an agreed methodology (MADRE) that takes into account effectiveness and cost-effectiveness evidence. GENESIS reports are a reference for the majority of hospitals in Spain.

The Andalusian and Catalan regions have shown greater commitment in the harmonisation of processes for the evaluation of new medicines. In 2002, the Andalusian HTA agency (AETSA) published a guide to the introduction of new medicines (GINF guidance) to facilitate the decisions on the inclusion of new medicines on hospital formularies [14]. In 2008, the Andalusian Health Service (SAS), established an advisory commission to harmonise the utilisation criteria for hospital medicines. The recommendations by the Andalusian Hospital Formulary guidance (GFTHA reports) are implemented by the SAS and hospitals within the region tend to endorse its guidance. GENESIS and GFTHA activities are coordinated to avoid duplication of assessments; and both use the GINF classification system to advise on the inclusion (categories C2, D, E) or non-inclusion (categories A, B, C1) of the medicine for hospital use.

Similarly, in 2008, the Catalan Health Service (Catsalut) established a harmonisation programme for pharmaceuticals (PHF) to guarantee equity in access to medicines and to improve efficiency in daily practice [15]. The PHF has a specific stream related to hospital medicines dispensed in outpatient care (PHF-MHDA), which operates with a technical committee (CAMHDA) and an executive committee (CFT-MHDA). PHF-MHDA follows a deliberative process based on CAMHDA assessment reports, which are conducted using GENESIS methodology, and relies on the consensus of both committees. Finally, the CFT-MHDA agrees on the level of restriction that applies to each medicine, which can be approved for a specific subgroup of patients, approved on individual patient basis or on a compassionate use regimen. Hence, PHF-MHDA final recommendations are mandatory in Catalonia and apply to every hospital in the region. While CatSalut is responsible for funding, the Spanish MSC) retains the competency of pricing and reimbursement (P&R) of medicines and, therefore, anchors regional policies to P&R agreements.

Lozano-Blázquez et al. (2015) compared the assessment processes for cancer medicines in England and Spain over the period January 2011–December 2013 [16]. They found that Spain produced more reports over that period compared to England (which is expected, as NICE only reviews a selection of technologies, prioritised according to some defined criteria); and also that NICE rejected a higher proportion of medicines. Lozano-Blázquez et al. (2015) provide some rationale for these differences; for example, more organisations conducting assessments in Spain are using more simple processes. The authors also point out that multiple regional or local reassessments may produce delays in access to cancer medicines [17].

The objective of this research was to study the impact of economic evaluation on the utilisation of cancer medicines in England and Spain. Although, the research cannot determine whether a centralised or regional approach to HTA is superior, by studying two countries with different approaches, we hoped to provide evidence that would be relevant to a wide range of European countries. Our aim was to extend the analysis of Lozano-Blázquez et al. (2015) in three ways: (i) by studying a longer time period, we expected our sample to include more medicines for which assessments had been made in both countries; (ii) by studying in more detail the assessment methods used, we hoped to shed more light on the relative consistency and rigour of the assessments in the two countries; and, more importantly, (iii) by analysing data on the utilisation of medicines over time, we hoped to explore what impact, if any, the assessments had on the access to, and use of, cancer medicines in the two countries.

## Methods

### Analysis of technology assessments

Data from both countries were extracted for all cancer medicines assessed during the period January 2008 to July 2015. We compared NICE reports with Spanish reports available at national level (e.g. IPT reports); regional level (e.g. GENESIS shared reports, CAMHDA reports and GFTHA reports); and hospital level (e.g. GENESIS reports from individual hospitals). Details were obtained of the medicines and indications studied, the methods used, the assessment (of clinical or cost-effectiveness) made and the resulting recommendations. NICE decisions were classified as recommended, restricted and not recommended in accordance with a previous analysis [18]. In addition, we recorded whether the recommendation was accompanied by a PAS.

For the GFTHA and GENESIS reports, we followed a similar rational to Lozano et al. and used the categories within the GINF classification to classify decisions as recommended (C2 and E); restricted (D) and not recommended (A, B, C1). CAMDHA decisions were classified as restricted (e.g. if approved for a specific subgroup or approved on an individual patient basis) and not recommended (e.g. compassionate use only). Positioning statements from IPT reports, and those GENESIS reports where the GINF system was not explicitly followed, were analysed by an oncologist author (JMB) who advised on the most suitable classification.

The recommendations made by NICE and the Spanish bodies were compared in a descriptive analysis. Similarly, within Spain, comparisons were made between different evaluation bodies in cases where more than one assessment existed.

### Analysis of data on medicine utilisation

We extracted monthly MIDAS (Medical Information Data Analysis System) data (made available by IQVIA) for the period from January 2011 to December 2016 measured in counting units (i.e. utilisation) and sales (i.e. expenditure in US dollars) for both countries. In addition, we extracted utilisation data for Catalonia (made available by CatSalut [Gerència de Farmàcia i del Medicament]), to assess whether regional usage was more responsive than national usage to recommendations made at the regional level.

Medicines were selected for this part of the analysis based on the following criteria (i) the medicine was for a single indication, or one major indication responsible for most of the usage; (ii) it had been assessed in that indication in both countries; and (iii) data were available for the full period of the analysis. Applying these criteria resulted in a sample of 11 medicines, representing the whole range of possible recommendations (e.g. recommended in both countries, not recommended in both countries, mixed recommendations across the two countries).

Volume results were presented in milligrams, in accordance with other existing analyses comparing the use of cancer medicines [19–21]. To determine the total utilisation in milligrams, the counting units were multiplied by the strength of the product (e.g. mg in a tablet, or mg per ml) in each case. To make calculations between both countries comparable, total utilisation in milligrams was estimated per 100,000 population. Population figures were obtained from The Office for National Statistics, the Instituto Nacional de Estadistica and the Instituto Estadistica Catalonia. In addition, since it is possible that the relative incidence of some cancers varies across populations, volumes (per 100,000 people) were adjusted by cancer incidence rates. Country-specific cancer incidence estimates were obtained from GLOBOCAN (data available for 2012). Throughout the paper, we use the incidence-adjusted analysis when discussing the impact of cancer recommendations made by NICE and Spanish HTA bodies on medicine utilisation in both settings.

The trend of medicines utilisation was then analysed to assess whether the reports published in either country had any noticeable impact. We used both descriptive statistics and a regression analysis to explore the impact of recommendations on medicine utilisation in England, Spain and Catalonia. In the latter, we analysed the 11 cancer medicines during 48 trimesters from 2011 to 2016 using a Poisson model with an exposure term; which allows us to explore the utilisation of a medicine depending on recommendation and expenditure, conditional on the incidence of the type of cancer the medicine is used for.

Quarterly country medicines expenditure *per unit* on each medicine was used as an explanatory factor of medicine utilisation, as a proxy of the cost of the medicine. Since expenditure might be endogenous (i.e. higher expenditure is also driven by higher utilisation), we tested the impact of excluding this from our model. In addition, we ran a secondary analysis to test the impact of considering the anticipated treatment cost (e.g. first year acquisition cost of the drug), as opposed to expenditure, in the results. Given the variability within the Spanish setting, this sensitivity analysis was restricted to the English model. The first year acquisition cost was estimated since this is the cost figure that would be foremost in the mind of local decision-makers deciding on how enthusiastically to adopt NICE guidance.

We recognise that there may be other costs (e.g. in administration of the drug, in treatment of adverse effects), as well as cost-offsets (e.g. discontinuation of an existing drug, if this is replaced by the new medication, or reductions in other non-pharmaceutical costs). However, most of these costs are likely to be relatively minor in practice. Where a first year treatment cost was cited by NICE, this was the figure used. When a cost was not cited, we have estimated treatment cost from information on the cost per dose and the number of doses given in the first year. In making these calculations, we assumed that patients would receive the prescribed dose until the end of their chemotherapy treatment regimen, or until the time they progress. Inevitably, these estimates are approximations, as (i) patients may have less than the prescribed dose due to lack of tolerance or adverse events, (ii) some drugs are administered based on the patient’s weight, (iii) there could be drug wastage, and (iv) doctors could continue treatment even when they progress, especially if there are no other therapies available. The costs excluded the confidential discounts through PAS schemes, as these go to the Department of Health, not the local decision-makers.

The data on recommendations used in the regression model were collected from NICE for England and from GENESIS reports for Spain; for those medicines where multiple GENESIS reports were available, we used the date of the first GENESIS report published as the reference for the analysis. For the purposes of this part of the analysis, recommendations were computed as either positive (‘recommended, restricted’) or negative (‘not recommended’). In Catalonia, medicines recommended for compassionate use were recorded as negative recommendations in the model.

We estimated a Poisson model with an exposure term^1^ that allowed us to take into account not just the count nature of the utilisation variable but also the different exposure each country had to the different types of cancers for which the medicines had an indication. The longitudinal models were estimated separately for England, Spain and Catalonia, taking the following form:

$${\text{Eu}}_{\text{dt}} = I{ \exp }\left( {\beta_{r} r_{\text{dt}} + \beta_{e} e_{\text{dt}} + \beta_{l} l_{\text{d}} + \beta_{m} m_{\text{dt}} + f_{t} + \varepsilon_{\text{dt}} } \right),$$

where *u*_dt_, *r*_dt_, *e*_dt_, and *m*_dt_ are, respectively, the medicine utilisation, the recommendation, the expenditure per medicine unit, and inclusion in the Cancer Medicine Fund (for England) of medicine d at time *t*. While *l*_d_ is a dummy variable for the use of medicine *d* in combination with another medicine. *f*_*t*_ is the time fixed effect. We use as an exposure term the incidence of the cancer for which the medicine has an indication.

Ideally, the model would capture two states of the world: the period before the recommendation, and the period after the recommendation, which can be either positive or negative. In order to understand the impact of the recommendation on the utilisation of the medicine, our first model used the positive recommendation as the basis for the analysis. Additionally, we conducted a second model using negative recommendation as the baseline. However, the negative model was difficult to compute due to the lack of utilisation data during the period before the negative recommendation. Consequently, we were unable to discriminate between no recommendation and negative recommendation for that period, which made results of the negative model difficult to interpret. In England, health authorities will approve the use of the medicine conditional on NICE recommendation. The situation is similar in Catalonia, where hospitals are not allowed to use the medicine before the PHF-MHDA issues its recommendation; whilst in Spain, if there is no recommendation, the medicine is still available through the central procedure.

Therefore, given the limitations of the data for the period before negative recommendation, we define recommendation as a positive recommendation, from NICE, GENESIS or PHF-MHDA, to understand the impact of that recommendation on the utilisation of a medicine.

The model coefficients are the proportionate change in the utilisation mean from one unit increase. We also present the marginal effect of a positive recommendation which is $${\raise0.7ex\hbox{${\partial {\text{Eu}}_{\text{dt}} }$} \!\mathord{\left/ {\vphantom {{\partial {\text{Eu}}_{\text{dt}} } {r_{\text{dt}} }}}\right.\kern-0pt} \!\lower0.7ex\hbox{${r_{\text{dt}} }$}} = \beta_{r} {\text{Eu}}_{\text{dt}}$$.

## Results

### Recommendations in technology assessment reports

In the period studied, there were 66 appraisals of cancer medicines published by NICE in England. A total of 11 (17%) medicines were recommended, 5 with a PAS. NICE restricted 39 (59%) medicines; 21 of which were accompanied by a PAS. Finally, 16 (24%) of medicines received a negative recommendation by NICE.

In Spain, there were 96 assessments undertaken by a range of bodies. We found a total of 17 IPT reports (41% Recommended; 53% Restricted; 6% Not Recommended); and 79 GENESIS reports (13% Recommended; 65% Restricted; 22% Not Recommended).

A total of 53 medicine–indication pairings were assessed in both jurisdictions. For these medicines, the rate of negative recommendations at the central level in England was higher than in Spain, where the MSC tended to equally recommend or restrict the use of the medicines. The pattern of negative recommendations of NICE and the SAS in Andalusia is very similar (43% vs 46%); whereas in Catalonia, most of the medicines (85%) were restricted based on clinical criteria.

### Methods employed in the assessment reports

The methods employed in all the NICE reports closely followed the Institute’s methods guidelines and consisted of a clinical assessment and a full cost-effectiveness study. In addition, where relevant, NICE’s ‘end of life’ guidance was employed, whereby the QALYs gained at end of life could be valued higher than ‘standard’ QALYs.

The methods employed in the Spanish reports were more varied, and were more focused on clinical efficacy and budget impact analysis. We found a significant level of duplication in the reports published within different regions for the same medicine. It is common for reports to be based on the same evidence and to cover the same time period; hence, the recommendations are similar. For example, the CAMDHA and GENESIS reports for afatinib were both conducted in 2014, both preceding the central IPT report issued for afatinib in 2015. Although both organisations recommend the use of the medicine, CFT-MHDA is more explicit regarding the clinical criteria that might apply to patients to access the treatment in Catalonia. Additionally, it places special focus on patient follow-up and the need to record the therapeutic response, in a specific registry (registre de pacients i tractaments de medicaments hospitalaris de dispensació ambulatòria).

Economic analyses conducted by GENESIS usually consisted of a summary of cost-effectiveness studies published by other organisations (e.g. NICE or the Scottish Medicines Consortium); incremental cost-efficacy estimates based on a non-systematic selection of clinical evidence; and an estimation of the economic impact to the hospital given the number of patients eligible for the new treatment. We found that the NICE assessment was published before the first Spanish report for five medicines out of the eleven medicines on which usage data were analysed. The reasons for such time differences in publication dates are varied and complex, but include the length of the evaluation processes and companies’ launch strategies, which could favour launching in the UK first. Therefore, a number of cost-effectiveness assessments by GENESIS, CAMDHA and GHTHA included NICE estimates as source of evidence for the Spanish recommendation (e.g. afatinib, aflibercept, bendamustine).

We also attempted to explore the impact of the different HTA approaches on time to access to cancer treatments. However, the complexity and differences between the pricing and reimbursement systems made comparisons difficult, even among regions in Spain. When marketing authorization is granted by the European Medicines Agency (EMA), the MSC initiates a procedure to decide on reimbursement of the new product on the national reimbursement list. After marketing authorization, the manufacturer first needs to go through some administrative tasks with the Spanish Medicines Agency (AEMPS), including obtaining a national product code, before the MSC initiates the procedure for pricing and reimbursement after the manufacturer application. However, different regions follow different publication procedures and timings; for example, in Catalonia, PHF-MHDA is not allowed to issue its recommendations until the price of the medicine is set at the central level, which might make PHF-MHDA appear slower compared with other regional HTA organisations that do not follow the same approach. Therefore, in Spain, any delay in access will be due to a combination of factors, rather than one specific issue, such as the time it takes to carry out the assessment itself, or the delay in the company’s application for pricing.

The situation in England is a little clearer, in that the (positive) recommendations made by NICE have to be implemented by the NHS within 3 months. However, in principle, patients can gain access to cancer medicines as soon as they are licensed, although in practice most NHS commissioners are likely to wait until the NICE report before allowing access other than in individual cases.

NICE appeared to be more restricted about the number of indications assessed and recommended for each medicine, although the CDF was in place to fund uses of medicines not recommended initially by NICE. In Spain, despite the absence of a specific mechanism to fund those medicines that were not recommended, we found that different regions did provide funding for different indications across the country.

### Descriptive analysis of medicines utilisation

Medicine usage over time was assessed to explore the level of impact of assessment reports in the two jurisdictions. We defined ‘impact’ to be ‘evidence of a change in the rate of usage within 3 months of the report being published’. We chose a period of 3 months, since it was unrealistic to expect reports to have an instant impact. Also, 3 months is the period given to the NHS in England to implement the recommendations made by NICE in its technology appraisals.

The 11 medicines and indications analysed for sales trends/usage were: afatinib (non-small-cell lung cancer), aflibercept (colorectal cancer), bendamustine (leukaemia), crizotinib (non-small-cell lung cancer, dabrafenib (melanoma), enzalutamide (prostate cancer), ipilimumab (melanoma), ofatumumab (leukaemia), pazopanib (renal cell carcinoma), vemurafenib (melanoma) and vinflunine (urothelial carcinoma). (Fuller details of the medicines studied, the HTA decisions made and the organisations making them are given in Appendix 1).

**Fig. 1:**
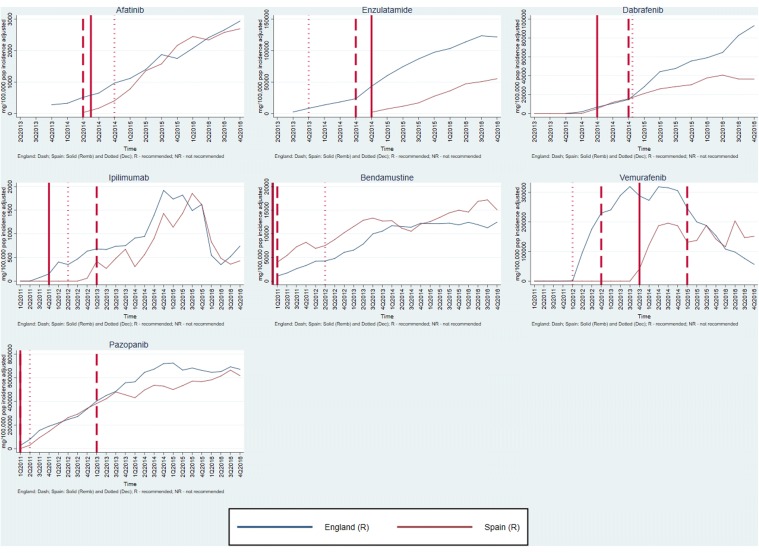
Medicines Recommended in both countries (UK and Spain usage)

**Fig. 2:**
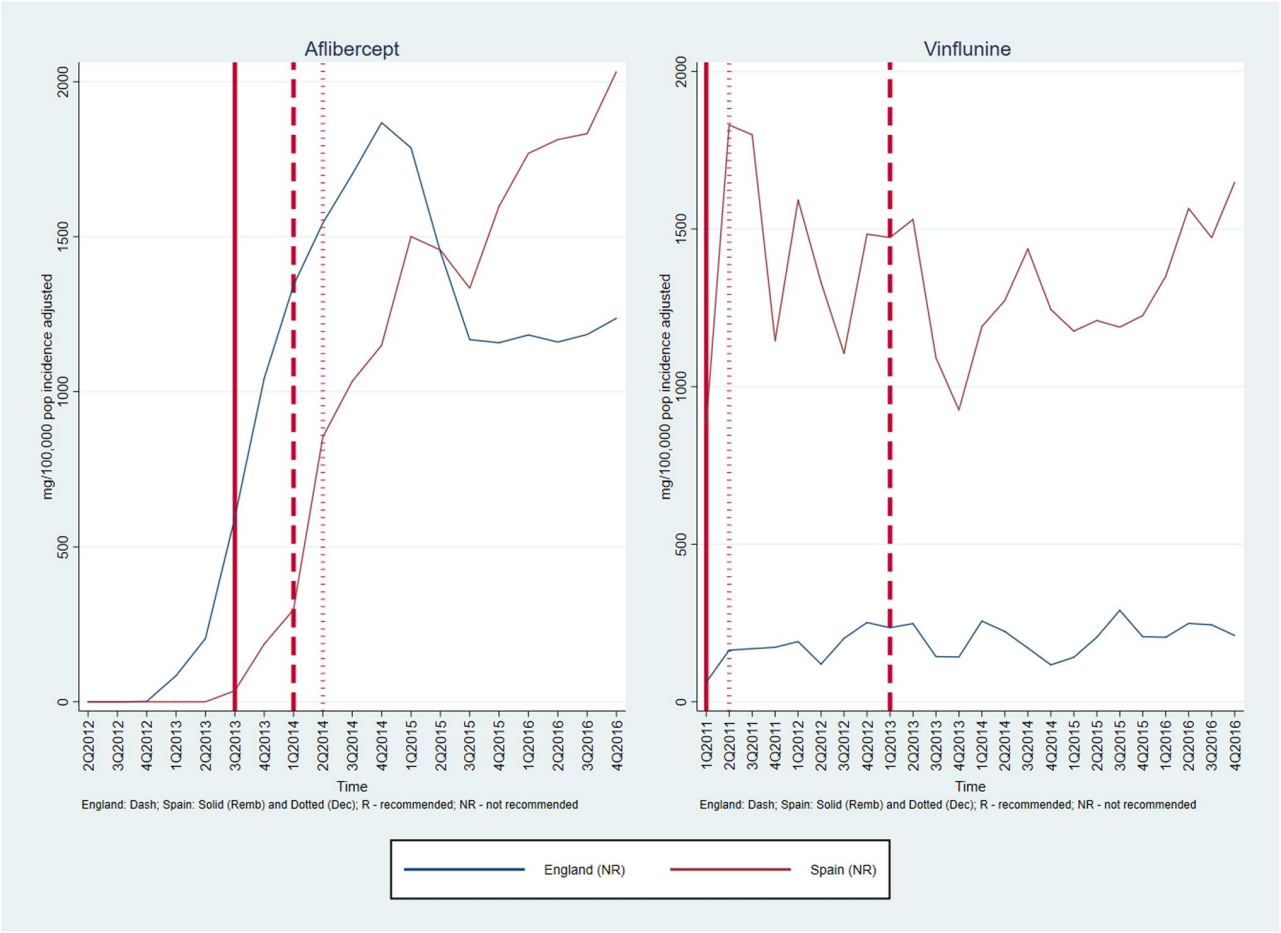
Medicines Not Recommended in both countries (UK and Spain usage)

**Fig. 3:**
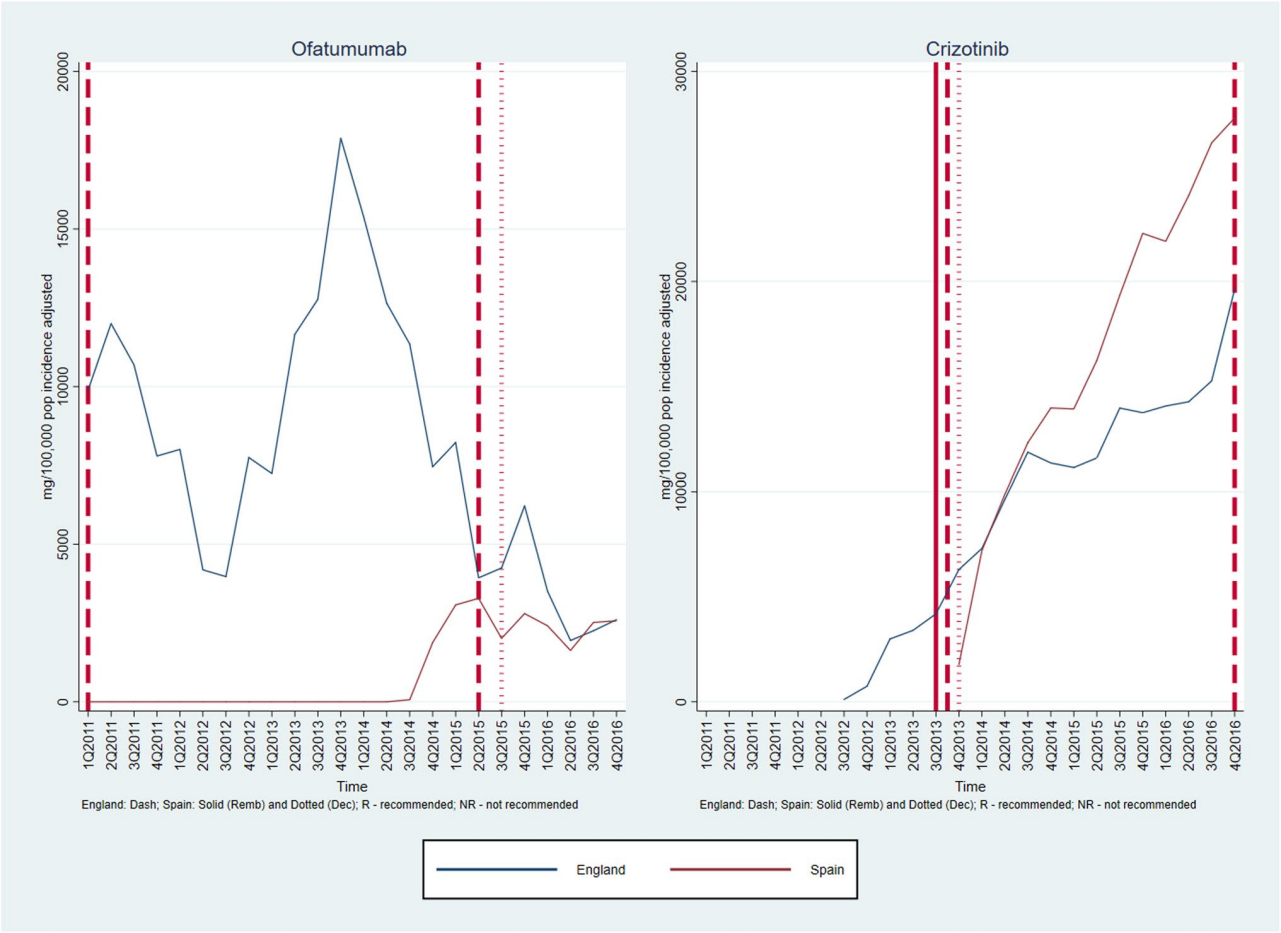
Mixed recommendations across the two countries (UK and Spain usage)

**Fig. 4:**
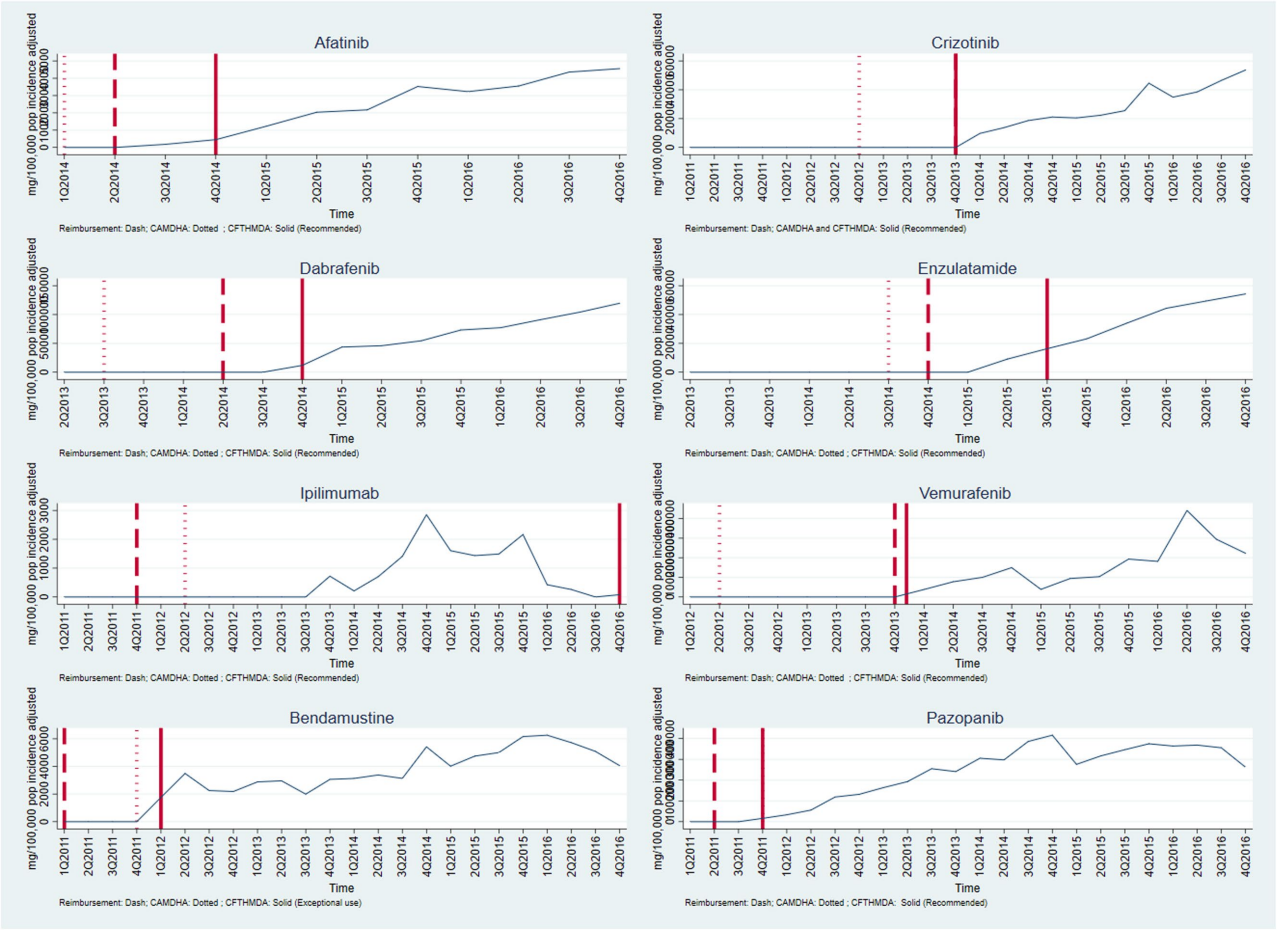
Medicines Recommended in Catalonia (Catalonia usage)

**Fig. 5:**
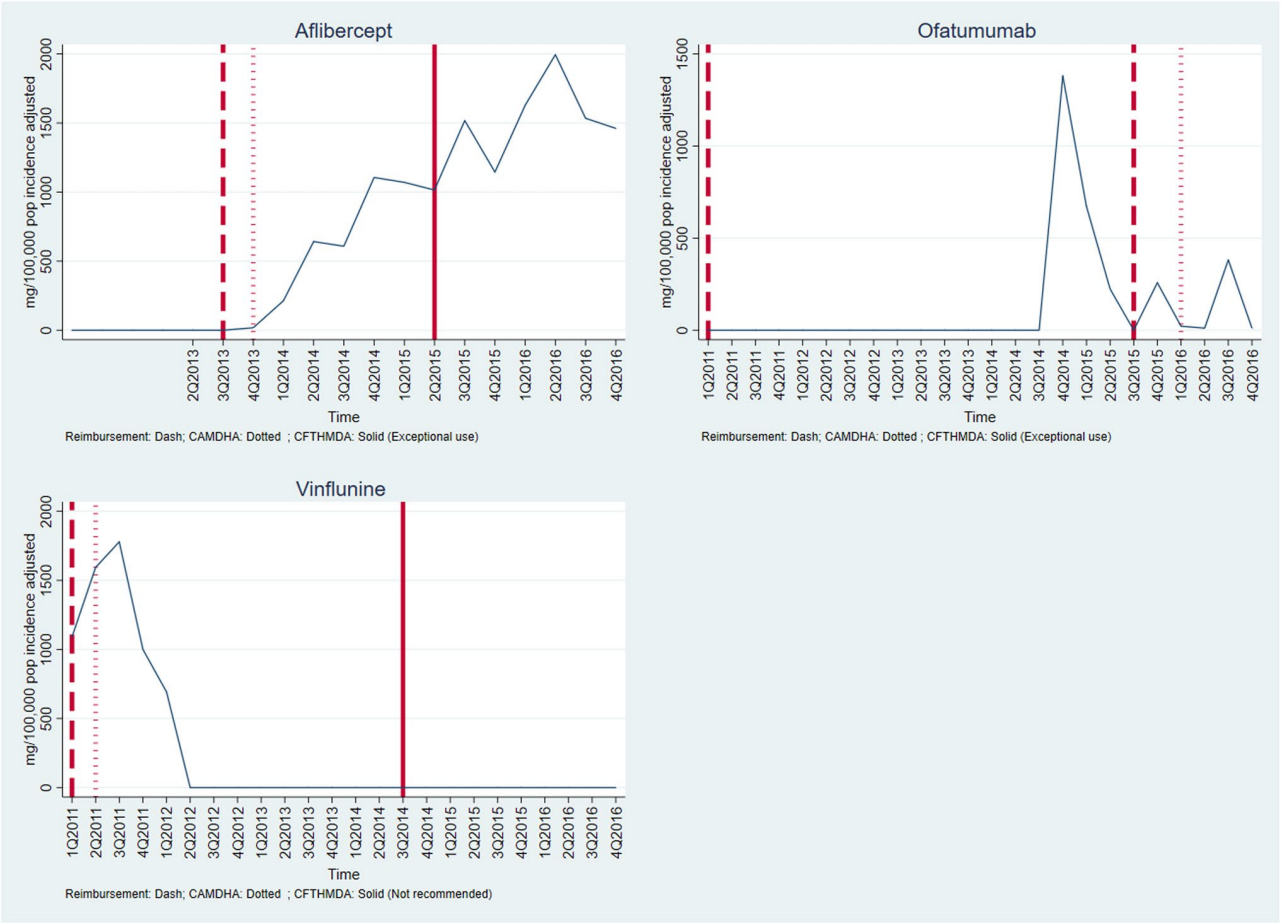
Medicines Not Recommended in Catalonia (Catalonia usage)

Graphs of medicines sales/usage over the period January 2011–December 2016 for England and Spain are given in Figs. 1, 2, 3. In each case, the dates of assessment reports in England (dash vertical line) and Spain (dotted vertical line), and P&R date by MSC in Spain (solid vertical line) are shown on the graph. For the purposes of the presentation, the medicines are discussed in three groups, according to the recommendations made in the two countries. Figures 4, 5 show the medicines sales/usage over the same period for Catalonia. These are presented separately as the data are from a different dataset.

Detailed interpretation of the trends for the various medicines is given in Appendix 2, but a few general points are made here. First, in considering the graphs for England and Spain (Figs. 1, 2, 3), it is clear that in both countries, there is some utilisation of all the medicines before any recommendation is made by an HTA agency. For most, but not all, of the medicines receiving a positive recommendation, there was an increase in the rate of utilisation following the recommendation, although in many cases there was already an upward trend in utilisation before the recommendation.

Secondly, although the HTA recommendations did have an impact, it is clear that other factors were influencing utilisation of the medicines concerned. One possible important factor was the launch of other medicines for the same patient group. This is one possible explanation for the usage of ipilimumab, which has the most complex pattern of those examined. The usage increased in both countries following a positive recommendation, but then fell away dramatically around the end of 2015. This could have been because of the launch of nivolumab, a competitor medicine in a new class (immunotherapy) around that time.

Thirdly, considering the medicines with negative recommendations in both countries, usage was never eliminated and in some cases did not fall. This emphasises the need for recommendations to be made in a timely fashion, since it is more difficult to control usage once the drug is already being prescribed.

Turning to the data from Catalonia (Figs. 4 and 5), the most striking finding is that in most cases, there was limited use of the medicines before the first recommendation. As in England and Spain (in general), usage of medicines increased following a positive recommendation. In situations where there was a negative recommendation, the usage fell more than in England and Spain, although in only one case (vinflunine) did this fall seem to be clearly attributable to the negative recommendation.

### Model results

Although the descriptive analysis of the 11 medicines gives some insights, it is clear that HTA guidance is not the only factor influencing usage, leading to differences in the impact of recommendations for individual medicines. However, we wanted to obtain an overall assessment of whether, on average, the guidance had a substantial impact. Therefore, we undertook a regression analysis to understand the impact of guidance on the overall utilisation of the 11 medicines in total in the two countries.

We observed that the utilisation and expenditure for each medicine varied across countries and over time. Also, the number of observations for each medicine differs across countries, since the medicine’s availably or utilisation might have begun at different points in time. Similarly, the recommendations from NICE, GENESIS and Spanish regional HTA organisations were issued at different times. For example, the NICE recommendation for bendamustine was in place for the full period analysed, since the decision was made in February 2011. In Spain, data on bendamustine usage were available from the first trimester of 2011; however, the GENESIS positive recommendation came in the third trimester of 2011. Hence, a recommendation for this medicine was in place for only 92% of our analysis period. Therefore, we chose a Poisson model, since this enables us to examine trends in the use of the medicines in relation to ‘exposure time’ to the guidance in each country.

The Poisson model results (Table 1) reveal that there is an increase of utilisation in England, Spain and Catalonia once the medicine has been positively recommended by NICE, GENESIS or PHF-MHDA, respectively. If there is a recommendation for the cancer medicine to be used in combination with another one, the utilisation decreases in England and Spain, but shows a small increase in Catalonia. In England, the CDF is used as a mechanism to fund the utilisation of medicines that are not recommended by NICE. In our model, there is a positive and significant coefficient of the CDF on the utilisation of those medicines not recommended by NICE, but funded by the CDF.

**Table 1:** Results of Poisson model

	England	Spain	Catalonia
Expenditure per unit (in 1000’s of USD)^a^	− 0.414*** (− 587.13)^b^	− 1.035*** (− 501.94)	− 1.308*** (− 149.59)
Used in combination with another medicine	− 0.336*** (− 225.41)	− 0.480*** (− 100.22)	0.0407*** − 4.98
CDF (not recommended medicines)	0.453*** − 83.96		
Positive recommendation by NICE	3.120*** (903.05)		
Positive recommendation by GENESIS/Spain		1.693*** (277.1)	
Positive recommendation by CAMDHA/Spain			4.892*** (139.36)

*T*-statistics in parentheses. * *p* value < 0.05, **p-value < 0.01 and ***p-value < 0.001

We have also included a time trend in all the models to capture the quarterly increase on cancer medicine utilisation

^a^Additionally, we run a secondary analysis to test the impact of considering the anticipated treatment cost (e.g. first year acquisition cost of the drug), as independent variable in the model for England

^b^The coefficient [(− 0.0108*** (− 238.26)] for the new independent variable [treatment costs (in £1000)] shows same results (e.g. the higher the treatment cost, the lower the drug utilisation is)

The coefficient for a CAMDHA positive recommendation (4.892) is higher than the one for NICE (3.120) and GENESIS (1.693). Also, inclusion in the CDF increases utilisation in England, but not as much as a positive recommendation by NICE (0.453). The time trend was included to capture the quarterly increase on cancer medicine utilisation in each region. In both countries, the units consumed (adjusted by 1000 population incidence) before a negative recommendation is quite low (9.2 in Spain versus 30 in England). Before a positive recommendation, the utilisation is lower in England than in Spain (597 versus 1458). After a positive recommendation, the units consumed (16 639 in England versus 11,310 in Spain) is more than 100 times bigger than after a negative recommendation (101 in England versus 182 in Spain).

The time effects capture the upper time trend of the cancer medicines utilisation. The medicine expenditure variable is negatively associated with the cancer medicine utilisation in all the three jurisdictions.^2^ Results for England are robust to sensitivity analysis, showing that, when using the anticipated treatment cost as an independent variable, conclusions do not change. Therefore, the use of treatment costs instead of expenditure unit cost does not change our main result, that a positive recommendation by NICE increases significantly the utilisation of cancer drugs. (Further details on the sensitivity analysis are given in Appendix 3). Our initial analysis plan stated that sensitivity analyses on the Spanish and Catalan settings would be conducted only if the sensitivity analysis on the English setting showed an impact in the results. Given that no impact was shown for England, sensitivity analyses using Spanish and Catalan data were not conducted.

The marginal effect of a positive recommendation is of 31,495,000 units for England, 6,791,000 units for Spain and 1,855,000 units for Catalonia. The magnitude of the marginal effect reflects not only the magnitude of the positive recommendation coefficient (reported above in Table 1) but also the average number of units utilised in each jurisdiction.

## Discussion

Our main finding was that positive HTA recommendations had an impact in both England and Spain. Within Spain, the impact was higher in Catalonia, a region with a strong track record of HTA.

The regional approach in Spain generated a higher number of HTA reports than in England. Although there was some duplication of effort in Spain, guidance was issued for 96 medicine/indication pairings, as compared with 66 in England. All the English reports followed NICE’s methods guidelines and produced an estimate of the incremental cost per quality-adjusted life-year (QALY) gained. However, some reports in Spain only addressed clinical effectiveness as opposed to cost-effectiveness, or just gave a summary of the results reported in an earlier NICE report. In addition, while the decision rule used to arrive at recommendations was clear in England, being based on the incremental cost per QALY gained, it was not clear in the Spanish reports.

Whether this suggests that the quality of reports is lower in Spain is a matter of judgement. Our view is that, while evidence on clinical effectiveness alone is useful, a decision on whether to use a drug should be based on clinical and cost-effectiveness. In addition, consistency in reporting is important if reliable comparisons of clinical or cost-effectiveness are to be made between different medicines. The variation in approach in Spain is understandable, given the range of organisations involved and the potential differences in their interests, or, in the case of IPT reports, a difference in the remit. In addition, despite the processes already in place to improve coordination between the national and regional levels, we observed a significant level of duplication of effort. It might be worth discussing ways in which communication is improved to promote a more efficient and systematic use of HTA resources.

The descriptive analysis on 11 medicines illustrated that, on some occasions, the recommendations in HTA reports appeared to impact on usage, but on other occasions not. Our main finding here is that usage is likely to be impacted by a number of factors and not just the HTA guidance. The factors probably include physicians’ own views on the effectiveness and cost-effectiveness of the various medicines and the availability of other medicines for the same patient group. For example, when we examined the consumption in both countries before a positive recommendation being released, we found that there was almost three times more consumption in Spain than in England, which on the one hand shows that recommendations have more effect in England, but, on the other hand, implies that patients in England were more likely to be denied early access to therapies that were eventually shown to be cost-effective.

It is not clear which situation is ‘better’ than the other. If one believes that the utilisation of drugs should be driven by the evidence of clinical and cost-effectiveness, the situation in England is to be preferred. If, on the other hand, one believes that patients should have access to promising new therapies unless these are shown *not* to be clinical or cost-effective, the situation in Spain is to be preferred. The reverse situation is observed for the consumption of medicines before negative recommendations. Although in both countries, there is usage of drugs that are eventually shown *not* to be cost-effective, the usage prior to negative recommendations was lower in Spain. One explanation is that, in England, the situation was complicated by the existence of the CDF, which at the time of our analysis provided funding for some medicines that had not received positive recommendations from NICE. However, although our sample was small, the impact of negative recommendations in England, where they are mandatory for the NHS, appeared to be stronger than that in Spain.

The regression analysis showed that, overall, positive recommendations had an impact on medicine usage in both England and Spain. The interesting finding here was that, although the strength of the impact on usage, as judged by the size of the coefficient, was higher in England than Spain, the strength of impact in Catalonia was the highest of all. As shown in the graphs, one possible explanation is that the lower effect of positive recommendation in Spain is driven by the fact that there is more utilisation before the recommendation; whereas in Catalonia, there is almost none. A positive recommendation still increases medicines’ usage in Spain, but probably increases it less because there was often substantial utilisation before the recommendation. This can be interpreted as an indication that a central system would allow for a better control of the medicines that are prescribed both before and after the recommendation. Whereas, a decentralised system such as in Spain, that was shown to be more variable with less clear guidance, showed lower control in the utilisation of medicines.

The finding of a strong impact of recommendations in Catalonia should be treated with some caution, since the data on medicine usage came from a different source, but they do lend some support to our hypothesis that local recommendations may have a greater impact. However, if true, it has implications for both the central and regional approaches to HTA.

For the central approach, the message is that, although centrally produced recommendations do have an impact, it still may be worthwhile putting effort into methods to encourage local adoption of the recommendations. For the regional approach, the message is that locally generated recommendations can have a strong impact, but that such an approach needs to be uniformly applied. For example, although there are 17 regions in Spain, only a minority of regions follow a structured HTA approach, with some regions having no locally produced reports at all. It appeared that in such a decentralised system, a substantial amount of decision-making is taking place at local level, especially in those hospitals where pharmacy committees work closely with HTA bodies to sustain a rational use and funding of medicines. This may be one factor contributing to the lower impact of guidance *overall* in Spain.

The evidence presented here relates to England and Spain. Inevitably, different features will apply in other countries, which limits the generalisability of our results. However, given similarities in HTA policies and processes in many other centralised and decentralised countries, we suggest that our conclusions may well be transferable. However, most importantly, our general findings suggest that investments in HTA, either centrally or regionally, can influence the usage of cancer medicines.

## Conclusions

The decision on whether HTA capacity is organised centrally or regionally depends on a number of factors, not least the level of autonomy granted to regions in the country’s constitution. This study shows that HTA recommendations made either centrally or regionally can impact upon medicine usage. However, if capacity is organised regionally, considerable effort may be required in coordination, to ensure consistent and rigorous assessments and adequate implementation of HTA findings.

## Electronic supplementary material

Below is the link to the electronic supplementary material.

Supplementary material 1 (DOCX 15 kb)

Supplementary material 2 (DOCX 19 kb)

Supplementary material 3 (DOCX 15 kb)

## Footnotes

1 We have used a Generalised Linear Models (GLM) framework to select the distribution and consequently, the model which has the best fit.

2 Models for England, Spain and Catalonia without the expenditure show very similar results (results are available by request to authors).
