# Supplementary material for: Does the use of health technology assessment have an impact on the utilisation of health care resources? Evidence from two European countries
Source: Eur J Health Econ. 2020 Feb 5;21(4):621–34. doi: 10.1007/s10198-020-01160-5 (PMC7214388; doi:10.1007/s10198-020-01160-5)
Supplement: Supplementary file 1 — Supplementary material 1 (DOCX 15 kb) [file 10198_2020_1160_MOESM1_ESM.docx]

**Appendix 1 – Medicines analysed for utilisation trends**

| **Medicine generic name and Indication assessed** | **EMEA licence**  **Date~** | |  | |  | **HTA – Effect on ACCESS** | | | | | |
| --- | --- | --- | --- | --- | --- | --- | --- | --- | --- | --- | --- |
|  |  |  | **Spain Nat. Code Date^** | | **Spain**  **Remb. Date†** | **England**   **NICE report decision** | **England Cancer Drug Fund** | | **Spain**  **Central (IPT)** | **Spain Regional (Organisation)** | **Spain Local (Comision Farmacoterapeutica- CF)** |
| **Afatinib**  Lung cancer (nsc) | | Sep 2013 | Oct 2013 | | Jun 2014 | R (PAS) Apr 2014 | NO | | Mar 2015 | R: PHF-MHDA ~ (Mar 2014 to Dec 2014) | R: CF Islas Baleares (Nov 2014) |
| **Aflibercept**  Colorectal cancer | | Feb 2013 | Feb 2013 | | Aug 2013 | NR Mar 2014 | YES | | NA | HR: PHF-MHDA ~ (Nov 2013 to June 2015) NR: GENESIS - May 2014 | CF Hospital Henares: Dic 2013 (PT) |
| **Bendamustine¬**  Leukaemia | | Mar 2010 | Jul 2010 | | Jan 2011 | R (PAS) Feb 2011/Jun 2015 | YES | | NA | R: PHF-MHDA ~  (May 2011 to Jan 2012) - (1L) R: GFTHA: Feb 2012 (1L) | ICO July 2011 (PT)  CF Hospital Uni. Virgen Rocio Apr 2013: NR (1L) / R (2L) |
| **Crizotinib**  Lung cancer (nsc) | | Oct 2012 | | Nov 2012 | Dec 2013 | NR (2L), Sep 2013  R (1L), Dec 2016) | YES (2L) | | NA | R: PHF-MHDA ~  (Nov 2012 to Dic 2013 (2L) R: GENESIS Sep 2014 (2L) R: PHF-MHDA ~ Aug 2016 to Feb 2017 (1L) | CF Hospital Gregorio Maranon Sep 2014 (PT) |
| **Dafratenib ¬** Melanoma | | Aug 2013 | | Sep 2013 | Jun 2014 | R (PAS) Oct 2014 | YES. | | Jun 2016 | R: PHF-MHDA ~ (Sep 2013 – Dec 2014/Dec 2016) |  |
| **Enzulatamide** Prostate cancer | | Jun 2013 | | Jul 2013 | Nov 2014 | R (PAS) Jul 2014 / Jan 2016 | YES | | July 2015 | R: GFTHA Oct 2013  R: PHF-MHDA ~ (July 2014 to Aug 2015) |  |
| **Ipilimumab**  Melanoma | | Jul 2011 | | Jul 2011 | Nov 2011 | REC (PAS) Dec 2012  July 2014  July 2016 | NO | | Apr 2015 | R (1L): GENESIS Aug 2012/2015  PHF-MHDA ~ (Apr 2012 to Apr 2013) – (R-2L)  (Jan 2014 to Dec 2016) – (EU-1L) | ICO July 2011 (PT)  CF Hospital Gregorio Maranon: May 2015 (R) |
| **Ofatumumab**  Leukaemia | | Apr 2010 | | NA | NA | NR Oct 2010  REC (PAS) Jun 2015 | YES | |  | UE: PHF-MHDA ~ (Sep 2015 to Mar 2016) | L’Agència d’Informació, Avaluació i Qualitat en Salut (AIAQS) – HTA report (2011) |
| **Pazopanib**  (renal cell carcinoma | | Jun 2010 | | Jul 2010 | April 2011 | REC (PAS) Feb 2011 /Feb 2013 | YES | |  | R: GENESIS (April 2011)  R: PHF-MHDA (Oct 2011) | CF Hospitsal Uni. La Fe (R) Mayo 2011 |
| **Vemurafenib**  Melanoma | | Feb 2012 | | Mar 2012 | Nov 2013 | REC (PAS) Dec 2012 | NO | | Nov 2013 | R: PHF-MHDA ~ (Apr 2012 to Dec 2013) | CF Hospital Gregorio Maranon  (R) Dec 2014 |
| **Vinflunine**  Urothelial carcinoma | | Sep 2009 | | Dec 2009 | Jan 2011 | NR Jan 2013 | NO |  | | NR: PHF-MHDA ~ (May 2011 to July 2014) | ICO April 2011 (PT)  CF Hosp. U Getafe, Nov 2011 -HR  CF Hosp. La Fe, Oct 2011 -NR |
