# Supplementary material for: Does the use of health technology assessment have an impact on the utilisation of health care resources? Evidence from two European countries
Source: Eur J Health Econ. 2020 Feb 5;21(4):621–34. doi: 10.1007/s10198-020-01160-5 (PMC7214388; doi:10.1007/s10198-020-01160-5)
Supplement: Supplementary file 2 — Supplementary material 2 (DOCX 19 kb) [file 10198_2020_1160_MOESM2_ESM.docx]

**Appendix 2. Detailed interpretation of the trends for the various medicines**

**Medicines receiving positive recommendations in both countries**

In England NICE issued positive guidance for afatinib (April 2014), enzulatamide (July 2014 and in January 2016 for another patient sub-group) and dabrafenib (October 2014). The graphs show a steady increase in the rate of usage over time, with a change in slope around NICE report, possibly in response to the positive NICE guidance. In the case of bendamustine and pazopanib, it is uncertain whether usage of the medicine was influenced by NICE guidance. NICE recommended use of bendamustine in February 2011 and also use of the medicine in combination therapy for another patient sub-group in June 2015. The graph shows a steady increase in use of the medicine until early 2014, when usage levels off. There is a slight suggestion of a small increase in use in late 2016, but based on only one observation. The same pattern is observed for pazopanib (recommended in February 2011 and updated in 2013); the graph shows a steady increase in use over the period studied, with use stabilizing in early 2014.

Ipilimumab represents one of the most complex patterns to interpret. NICE issued two positive recommendations, in December 2012 and July 2014, for different patient sub-groups. Then, in July 2016, NICE issued another positive recommendation for use of the medicine in combination with nivolumab. The graph shows a steady increase in use over time, the largest change in the rate of use being in the middle of 2014, around the time of the second positive appraisal. One possibility is that the drop in use in 2015 could have been influenced by the licensing of nivolumab around that time, although it was not recommended by NICE until February 2016. Nevertheless, the resurgence in the use of ipilimumab in mid-2016 may be attributable to the positive NICE guidance for its use in combination with nivolumab.

It is possible that the complex pattern of usage for some medicines is a result of other medicines being recommended for the same patient group. For example, vinflunine was recommended by NICE in December 2012, with an update in January 2015. .In addition, NICE recommended several other medicines for the same patient group within a similar timeframe; dabrafenib in October 2014, ipulimumab in July 2014. The graph shows an increase in the use of vinflunine from launch, which continued after the positive NICE appraisal. However, usage plateaued in 2013 and then began to fall in 2015. This may be because of the other medicines being approved for the same patient group.

In Spain, different HTA bodies also issued positive guidance for these medicines. In all cases we observe that the graphs show usage beginning around the time the MSC decided on pricing and reimbursement, which is clearest for enzulatamide, bendamustine and pazopanib. The MSC decided on the reimbursement for enzulatamide in November 2014. There are two GENESIS reports; positive guidance by the GFTHA (Andalucia) in October 2013 (one year before reimbursement was agreed at national level) and a second positive recommendation by CAMDHA, which conducted an assessment in July 2014, subsquently published in January 2015 (e.g. after reimbursement by MSC). Then, in July 2015 the MSC issued an IPT report for the medicine at central level. The graph shows a steady increase in the rate of usage from reimbursement, with a change in slope around the time of the IPT report, possibly in response to its positive guidance.

Bendamustine was assessed for the three different indications in Catalonia in July 2011 (recommended for all indications by CAMDHA and assessed by the ICO). In was also assessed in October 2011 in Andalucia (recommended for one indication and not recommended for a second one). In April 2013 another GENESIS report (Hospital VR in Andalucia) issued a negative recommendation for a third subgroup of patients. There is no central positioning report for this medicine. Nevertheless, we can observe a steady increase of utilisation from the time the medicine was included in the reimbursement list; however, it is not clear whether the usage of the medicine was influenced by regional guidance. All Spanish assessments included a NICE assessment as source of evidence in its reports, hence it might be possible that the increase rate of usage in Spain was also influenced by NICE’s positive guidance.

Regarding ipilimumab, a similar pattern to England is also observed in Spain; hence it is possible that the interaction of other medicines have certainly an impact in the usage of specific medicines. In the case of afatinib and dabrafenib, there are both central (IPT report March 2015 and June 216 respectively) and regional guidance by CAMDHA. In both cases we observe a change in slope around the time of the IPT report, therefore it is likely that central level guidance had an influence.

**Medicines receiving negative recommendations in both countries**

In England, NICE issued a negative recommendation on the use of aflibercept in March 2014. In the fourth quarter of 2014, the graph shows a sharp fall in usage, resulting in a constant lower level of use. This suggests a response to the NICE recommendation, albeit somewhat delayed. The fact that some patients remain on the medicine is not inconsistent with the NICE guidance, as it is common for NICE to allow patients already on the medicine to continue therapy. In Spain, there was no central guidance for this medicine. Two recommendations were issued at the regional level. CAMDHA endorsed a highly restricted use in Nov 2013, whilst GENESIS group issued a negative recommendation in May 2014. However, the pattern in usage shows a steady increase over time, suggesting that usage of aflibercept did not respond to the HTA guidance in Spain.

NICE issued a negative recommendation on the use of vinflunine in England in January 2013. The graph shows that there was modest use before 2013, but no increase following the report, suggesting that only the patients receiving the medicine at the time of the appraisal remained on the medicine. Therefore, it seems as though the assessment report contributed to a lack of increase in use, although clinicians may not have used the medicine in any case if they perceived it to have limited benefits. In Spain, the trend of the usage showed in the graph is again inconsistent with the negative recommendation by CAMDHA in May 2011, being generally higher than the usage in England and showing a slight increase after the report.

**Medicines with mixed recommendations in the two countries**

In England, NICE issued a negative recommendation on crizotinib in September 2013 (first dash line) and then, based on a reconsideration, issued a positive recommendation (second dash line) in December 2016, providing a PAS was offered. The graph shows an increase in the use of the medicine, with a slowing down in the rate in 2014. This indicates some response to the NICE recommendation, but the usage continued to grow, albeit at a lower rate, possibly because the medicine was funded through the Cancer Medicines Fund from June 2016. In Spain, we also observe an increase in the use of crizotinib following reimbursement by the MSC in Dec 2013, possibly because the medicine was also recommended at the regional level both by CAMDHA and a GENESIS report. Therefore, some response is shown to regional guidance in Spain.

Regarding ofatumumab, NICE issued a negative recommendation in October 2010, followed by a positive recommendation (with a PAS) in June 2015. The graph shows a dip in usage in early 2011, followed by a large increase in use in 2013. Use then falls from early 2014. In Spain, CAMDHA only approved the use of this medicine on exceptional use. The graph shows a limited use over time, although its usage increases in the second quarter of 2016. Therefore, changes in the use of this medicine appear to be influenced by factors other than NICE or regional Spanish guidance.

**Medicines in Catalonia**

One of our hypotheses was that usage of health technologies might be more responsive to local guidance. Therefore, it is interesting to examine the usage of medicines in Catalonia in response to PHF-MHDA guidance. We were able to achieve this by using data on medicine usage made available to us by CATSALUT.

In Catalonia, the assessment process by PHF-MHDA is indirectly influenced by the price and reimbursement (P&R) conditions from the MSC, as the P&R conditions need to be known in order to be incorporated into the assessments to inform final deliberations. In order to reflect this fact we included three dates in the graphs: reimbursement date (dash line), CAMHDA assessment (dotted line) and CFT-MHDA final recommendation. As shown in the graphs, the delay in the P&R negotiations of afatinib and dabrafenib would explain longer evaluation times for these medicines in Catalonia. The delay is longer in the case of crizotinib and vemurafenib, which technical reports were on standby for 13 and 20 months respectively, waiting for a P&R agreement.

The PHF-MHDA process for the evaluation of afatinib started on March 2014 (CAMHDA report), R&R conditions by the MSC became available in June 2014, finally the CFT-MHDA committee issued a positive recommendation in December 2014. The graph shows a slow increase in the use of the medicine following P&R agreement, growing after CA-HDA report, and increasing the rate from December 2014 onwards in response to CFT-MHDA final recommendation. The same pattern is observed for crizotinib, dabrafenib, enzulatamide and pazopanib, where we note a strong increase in consumption following positive guidance by PHF-MHDA.

In the case of bendamustine and pazopanib, it is uncertain whether usage of the medicine was influenced by PHF-MHDA guidance. For ipilimumab, we observe the same pattern we saw in England and Spain. Hence it is possible that the introduction of nivolumab had an impact in the Catalan setting as well.

PHF-MHDA issued a negative recommendation on the use of aflibercept and ofatumumab. However, the graphs do not suggest a response to these recommendations. In contrast, a sharp decrease in usage of vinflunine is observed following its negative recommendation (e.g. exceptional use only) in April 2011, suggesting strong response to the PHF-MHDA recommendation.
