# Supplementary material for: Does the use of health technology assessment have an impact on the utilisation of health care resources? Evidence from two European countries
Source: Eur J Health Econ. 2020 Feb 5;21(4):621–34. doi: 10.1007/s10198-020-01160-5 (PMC7214388; doi:10.1007/s10198-020-01160-5)
Supplement: Supplementary file 3 — Supplementary material 3 (DOCX 15 kb) [file 10198_2020_1160_MOESM3_ESM.docx]

**Appendix 3. Sensitive analysis using treatment costs for the English setting.**

| Poisson model for England | | | |
| --- | --- | --- | --- |
|  | Main model | Sensitive analysis |  |
|  | Model with expenditure | Model with treatment cost |  |
| Expenditure (per unit) | -0.414*** |  |  |
|  | (-587.13) |  |  |
| Treatment cost (in £1000) |  | -0.0108*** |  |
|  |  | (-238.26) |  |
| Used in combination with another medicine | -0.336*** | -0.650*** |  |
|  | (-225.41) | (-409.93) |  |
| Positive recommendation by NICE | 3.120*** | 3.335*** |  |
|  | (903.05) | (982.90) |  |
| CDF (not recommended medicines) | 0.453*** | 1.002*** |  |
|  | (83.96) | (186.27) |  |
| Observations | 226 | 226 |  |

When we use the treatment cost instead of the unit cost, the impact of a positive recommendation is almost the same. The coefficient treatment cost is smaller than the drug expenditure by unit because the treatment cost is 1.5 times higher. Therefore, the usage of treatment cost instead of expenditure unit costs does not change our main result that a positive recommendation by NICE increases significantly the utilisation of cancer drugs.
